# Flavoured Vaping Products in Tobacco Harm Reduction: A Regulatory Perspective

**DOI:** 10.7759/cureus.89196

**Published:** 2025-08-01

**Authors:** Ian M Fearon, Matthew Stevenson, Thomas Nahde

**Affiliations:** 1 Scientific Research, whatIF? Consulting Ltd., Harwell, GBR; 2 Group Science and Regulatory Affairs, Imperial Brands PLC, Bristol, GBR; 3 Group Science and Regulatory Affairs, Reemtsma Cigarettenfabriken GmbH, Hamburg, DEU

**Keywords:** e cigarettes, electronic vaping products, flavours, public health, tobacco harm reduction

## Abstract

Reducing the harms associated with cigarette smoking is a global public health priority. Both the individual‑ and population‑level health impacts of smoking can be reduced by novel products which deliver nicotine, but the use of which decreases user exposure to the harmful chemical toxicants responsible for smoking‑related disease. Electronic vaping products (EVP) heat a flavoured liquid, most commonly containing nicotine, to generate an inhalable aerosol. Smokers who switch exclusively to using EVP reduce their exposure to the harmful toxicants found in cigarette smoke, and increasing evidence supports that EVP can help smokers quit smoking and support tobacco harm reduction (THR) efforts. However, concerns are being raised regarding the use of EVP among those for whom they are not intended, particularly youth, which could mitigate their THR potential. Some of this concern is attributed to the use of non‑tobacco flavours in EVP, and there have been many calls to ban the use of such flavours. The aim of this review is to examine the recent scientific literature concerning non‑tobacco flavours in EVP, focussing on the potential toxicological impact of these flavours, whether flavours affect abuse liability/dependence potential and whether they support switching away from cigarette smoking, unintended use of flavoured EVP, and the impact of existing bans and the effect of regulation. Overall, we find that non‑tobacco flavoured EVP are not of a greater risk to health and have no greater abuse liability than tobacco flavoured EVP. There is some evidence that flavoured EVP may support switching among adult smokers, while unintended use may be experimental and may not translate into long‑term use or act as a ‘gateway’ into smoking. Current flavour bans have been largely ineffective in modifying use behaviour and may have unintended consequences such as increasing smoking prevalence and promoting illicit trade. Overall, the population‑level THR benefit of flavoured EVP, in terms of reducing smoking rates, largely outweighs the risks associated with unintended use. Maintaining flavour diversity is an important consideration when putting in place regulatory frameworks that aim to maximise the switching benefit for adult smokers and improve overall population‑level public health.

## Introduction and background

Reducing the harms associated with cigarette smoking is a global health priority to help lower the estimated annual seven million preventable global deaths associated with smoking [1]. While nicotine in cigarette smoke is not harmless and is addictive, it is not the primary cause of the harmful effects of cigarette smoking [2-5]. Instead, smoking‑related diseases are caused by smokers inhaling chemical toxicants, which are formed during the combustion and pyrolysis of tobacco [6]. Around 7,000 individual chemicals have been identified in cigarette smoke [7], and some of these have been defined by the United States (US) Food and Drug Administration (FDA) as ‘Harmful or Potentially Harmful Constituents' (HPHCs) which are linked to cardiovascular disease, respiratory disease, lung cancer, and reproductive/developmental toxicity [8].

**Figure 1:**
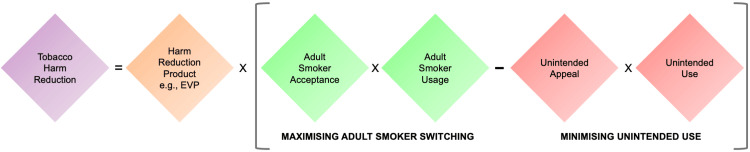
Factors determining the tobacco harm reduction potential of novel tobacco and nicotine products EVP: electronic vaping product

In 2001, the US Institute of Medicine (IoM) published its report on ‘Clearing the Smoke-the Science Base for Tobacco Harm Reduction’ [9,10] in which it outlined how novel tobacco and nicotine products could 'lower total tobacco-related mortality and morbidity even though use of that product may involve continued exposure to tobacco-related toxicants'. This suggests that while a significant proportion of individuals will continue to use tobacco and/or nicotine, tobacco harm reduction (THR) can be achieved by either decreasing the risks associated with tobacco/nicotine use, decreasing users’ consumption, or decreasing the prevalence of use. This gives rise to the concept that both the individual‑ and population‑level health impacts of smoking can be reduced by novel products which deliver nicotine but the use of which reduces user exposure to the harmful chemical toxicants responsible for smoking‑related disease. Support for such an approach to THR is increasing, and several global health authorities now advocate for switching to using novel nicotine products which reduce exposure to harmful chemical toxicants and, therefore, reduce risks to health among individuals who smoke [3,4,11-13]. Calls for other global health authorities to make THR a strategy central to promoting public health have also been made [14-16]. Numerous factors are thought to be involved in determining the population‑level THR potential of novel tobacco and nicotine products (Figure 1). Novel products must expose users to lower levels of toxicants than those found in cigarette smoke and therefore possess THR potential. They must be sufficiently accepted and used by adult smokers as a complete substitute for cigarettes or as a means to substantially reduce cigarette consumption. In addition, the appeal of novel products among those for whom they are not intended must be minimised, particularly among susceptible populations such as youth and never smokers, in which novel products could pose an initiation or reinitiation risk [17,18].

Electronic vaping products (EVP), also termed e‑cigarettes or electronic nicotine delivery systems, are battery‑powered devices which heat a flavoured liquid solution, most commonly containing nicotine, to generate an inhalable aerosol [19]. EVP pose a potentially reduced health risk compared to cigarette smoking since their aerosols contain fewer and substantially lower levels of toxicants responsible for smoking‑related disease [20-22]. This gives rise, among those smokers who switch exclusively to EVP or substantially reduce their cigarette consumption, to reduced toxicant exposure [23-27]. Increasing amounts of evidence coming from randomised controlled trials and observational studies suggest that EVP can help smokers quit smoking [28-34], and the increasingly widespread availability of EVP has been associated with reductions in smoking prevalence at the population level [35-37]. For example, analysis of US National Health Interview Survey (NHIS) data found that post‑2007, a time at which EVP began to be marketed, adult smoking prevalence was significantly lower than expected based on pre‑EVP era trends. These declines grew as EVP use prevalence increased and were larger in subpopulations with higher e‑cigarette use, especially younger adults aged 18 to 34 [35]. Furthermore, a study which examined data from countries with historically similar smoking trajectories but differing current electronic cigarette regulations (United Kingdom (UK) and US vs. Australia, where sales of nicotine‑containing EVP are banned) found that the decline in smoking prevalence seems to have been slower in Australia than in the UK overall and slower in Australia than in both the UK and the US among both young people and those in lower socioeconomic groups [36]. Both of these findings point to the THR potential of EVP. This may, however, be mitigated by the use of EVP among unintended populations such as never smokers and youth [17,18,38], and youth EVP use has been reported to be rising in many countries including those in Latin America [39], in Asia [40], and in Africa, [41], as well as in the UK [37], and in the US [42] although youth EVP use in the US does appear to be declining in more recent years [42]. Because of these increases in youth EVP use, an increasingly bright spotlight is being shone on the use of non‑tobacco flavours in EVP, including fruit, sweet, and dessert flavours. It has been proposed that non‑tobacco flavours may be more appealing to unintended users, including youth, than tobacco flavours [43-47], and may also elevate health risks associated with EVP use [43,44,48-50]. This has led to proposals to restrict or ban the use of non‑tobacco flavours in EVP [43-45,51,52]. However, such proposals may have implications for THR. The aim of this review is to examine the recent scientific literature on EVP flavours, with a view to exploring whether flavours pose an increased health risk, and whether their availability is supportive of, or detrimental to, population‑level public health and THR strategies.

## Review

Do EVP flavours pose an increased health risk?

Any potential health risk assessment of EVP flavours can be determined either by undertaking desk‑based quantitative risk assessments (QRAs) for individual flavour ingredients [53], by assessing the levels of toxicants in EVP aerosols and using the data generated to inform toxicological QRAs, or by conducting laboratory toxicological studies. Using analytical chemistry approaches with flavoured EVP aerosols, studies have reported the detection of metals [54-59], though in some of these studies, there was a large degree of variability between different device types and brands [58,59]. Furthermore, in two studies, tobacco‑flavoured aerosols contained the highest metal concentrations [54,60], while in a further study, nickel in EVP aerosols was similar for tobacco‑ and mint-/menthol-flavoured EVP from one brand, lower in mint-/menthol-flavoured EVP aerosols from two brands, and higher in mint-/menthol-flavoured EVP aerosols from another brand [58]. These studies may be hampered, however, by methodological flaws, including generating EVP aerosol under unrealistic experimental conditions, which cause coil overheating or using inappropriate airflow across the coil [61]. Other studies have identified volatile organic compounds [62], carbonyls including formaldehyde, acetaldehyde, acetone and acrolein [63], other HPHCs [64], and potentially harmful flavour adducts formed by chemical interactions with the e‑liquid diluents propylene glycol (PG) and vegetable glycerine (VG) [65,66], in EVP aerosols. It is notable regarding these toxicants that few studies have directly assessed differences between flavoured and unflavoured or tobacco‑flavoured products in a like‑for‑like comparison (i.e., assessing tobacco and non‑tobacco flavours used under the same conditions and in the same device), and between‑study comparisons are challenging due to differences in experimental study design as well as the products used to generate EVP aerosol. Further studies are required to address this issue and to provide specific information concerning whether the use of flavours in EVP does, in fact, change aerosol toxicant levels. Likely though, it is not a simplistic matter of one flavour type causing greater toxicant release into the aerosol than another type, but a more complex one involving flavour/device interactions as well as the individual flavour components used by different manufacturers within their flavours. 

While the findings from EVP aerosol toxicant assessments may appear concerning at first sight, for each of these studies, QRAs informed by the levels of the toxicants detected, as well as estimating consumer exposures and doses at which toxicological effects are known to occur, were not conducted. In this regard, it should be noted that accurate assessment of any potential health risks is a multi‑step procedure informed by identifying the hazard (toxicants), assessing a dose response, assessing the likely exposure level, and characterising the risk to determine whether it is acceptable. Carrying out such multi‑step procedures is of critical importance since sensitive analytical equipment may detect potential toxicants at levels lower than those which would be of concern to health under typical patterns of EVP use, particularly for metals [67]. It is of equal importance, given that regulatory proposals aim to restrict non‑tobacco-flavoured EVP, to use tobacco-flavoured e‑liquids as experimental controls, and also to generate EVP aerosols under realistic conditions which equate to typical human puffing behaviour [68] and use realistic EVP power outputs which do not cause EVP coil overheating and the phenomenon known as 'dry puffing' [69-73]. In many of the studies mentioned, none of these steps was undertaken. Of additional importance, given that the intended population and the overwhelming majority of EVP users are former smokers [74,75], using cigarette smoke as an experimental control is also fundamental to inform relative risk assessments. Furthermore, some studies have assessed the impact of exposure to e‑liquids on toxicological endpoints using in vitro assays [76-84]. Such experimental models do not reflect the actual route of exposure among EVP users, which is inhalational. Therefore, studies assessing toxicological impacts of flavoured EVP should use models which reflect the exposure route and expose cells in vitro to EVP aerosol or aerosol extracts [85], and again, the use of puffing regimens which reflect typical human use is paramount. In vivo animal studies have also been conducted to assess the toxicological impacts of flavours, and their use is valuable for providing evidence of biological plausibility. However, extrapolating outcomes to effects in humans is a limitation of in vivo models, and they may not accurately predict human exposure and disease risk [86]. There are also ethical concerns around tobacco product testing in animals [87,88]. Regarding methodological limitations of in vivo models, those models which assess the impact of flavours administered orally through drinking water may not reflect EVP user exposure to flavours contained within the aerosol, and as seen in some studies, may overexpose animals to the flavouring agent [89]. When examining findings from in vivo studies which have, to the extent possible, mimicked human exposure, EVP aerosol was found to be less toxic than cigarette smoke in models assessing inflammatory, respiratory, cardiovascular, gastrointestinal and renal endpoints [76,90-92], and little or no between‑flavour toxicologically‑relevant differences were observed [76,90,92]. Such findings concur with many other in vitro and in vivo studies [50,56,93-100]. In studies which have observed between‑flavour differences, these may be confounded due to flaws in the experimental design. For example, due to the addition of flavourings at levels exceeding those found in e‑liquids [76,82,101,102], cultured cells in vitro may have been exposed to a much higher level of flavouring than would occur during human aerosol exposure. Additionally, observations of flavour effects in aerosol‑exposed in vitro models may be due to misuse of recommended puffing regimens, such as the CORESTA method number 81 [103]. This method stipulates a three‑second puff duration with puffs taken every 30 seconds, which has been used in some studies, but for excessive numbers of puffs and with no indication of puff volume [76], both of which mean that the puffing regimen used deviated significantly from a recognised standard and typical EVP user topography. Other studies have used EVP aerosol captured by bubbling through cell culture media or phosphate-buffered saline, with some vaporising significantly more e‑liquid than would be expected during typical use by an EVP user over the same period [84]. Such issues with dosimetry, along with other potential issues with the use of in vitro models [104], must be addressed in order to improve the validity of these models. This must include standardisation and rigorous reporting of the aerosol generation process [104].

While many studies have suggested a lower in vitro and in vivo toxicological impact of EVP aerosol compared with cigarette smoke, and only minor between‑flavour differences, we should not disregard the fact that some flavour additives or ingredients have the potential to give rise to toxicological concern when used in EVP liquids. Various studies described above have demonstrated the potential for impacts on human health of individual flavours, although many of these studies are hampered by the experimental flaws we described previously such as the lack of a relevant control exposure, the use of experimental models not relevant to assess the potential impact of EVP use (e.g. liquid and not aerosol exposure or inappropriate experimental models used), and the lack of a rigorous stepwise health risk assessment or QRA. For example, studies in a variety of in vitro and in vivo experimental models have reported potential toxicological effects of cinnamaldehyde (a cinnamon flavouring) [79,81,105], vanillin (a sweet, vanilla flavouring) [80,106-108], benzaldehyde and other aldehyde flavourings [66,81,109], ethyl butanoate and diacetyl (butter‑like flavourings) [110,111], eugenol (a spicy/clove flavouring) [112], hexyl acetate (an apple flavouring) [112], and ethyl esters (a citrus flavouring) [113]. There is also the potential for individual flavour ingredients to undergo chemical reactions with one another and with other ingredients (e.g. PG and VG) to form additional chemicals which may be of toxicological concern [49,65,66]. However, it is important to stress that due to the large number of potential e‑liquid flavour ingredients and also due to the potential for both antagonistic and synergistic effects of individual flavour ingredients, a pragmatic and holistic approach to testing may be required. This could include, for example, testing of either whole e‑liquid or aerosol, which, in addition to assessing a relevant exposure article, gives the benefit of capturing any ‘de novo’ formation of constituents formed upon mixing or heating/vaporisation. Another potential approach is to use a ‘flavour toolbox’ approach in which individual flavour ingredients are assigned to groups based on structural, metabolic and toxicological similarity (read‑across), from which flavour group representatives are used to create mixtures for laboratory testing [85,91,114]. Such an approach has been used by the European Food Safety Authority, among others, to assess the toxicity potential of food flavour ingredients [115,116] and could be applied to EVP.

Taking into account the literature reviewed above, overall, there is no strong indication that non‑tobacco-flavoured EVP aerosols pose a greater health concern than tobacco‑flavoured aerosols. Studies assessing potential health impacts of flavour components added to EVP liquids are challenging, and extreme care needs to be taken when designing experiments to assess this potential. Given the potential for the inclusion of certain flavourings to give rise to health concerns, rigorous QRAs taking a stepwise approach using both desk‑based computational and laboratory‑based techniques must be included in the overall process of EVP liquid development. Such assessments should identify the hazard, take into account a relevant route of exposure, include appropriate control or reference exposures, use models appropriate to reflect this exposure including the use of 3D tissue models [76,97,117-119], and where possible and relevant, take into account any dose‑response and exposure levels during typical use in order to fully and accurately characterise the risk. Additionally, studies should set EVP device power conditions according to those used by EVP users, use puffing regimens that approximate normal use patterns and topography, assess the potential for the transfer of flavours from the liquid into the aerosol, and determine the potential for chemical interactions in ingredient mixtures [85,117,120]. Additionally, when potential toxicants are detected in analytical chemistry studies, the exposure level must be taken into account in order to accurately ascertain whether a human health risk exists [121]. Appropriate use of such methodologies will serve to reduce the potential for adverse effects of flavouring inclusion and help to optimise the THR potential of EVP. Fundamentally, there is an onus on manufacturers to incorporate a rigorous product stewardship approach, such as that proposed by the UK Committee on Toxicity of Chemicals in Food, Consumer Products and the Environment (COT) [122], into the development of e‑liquid formulations to ensure product quality and consumer safety. Such an approach to e‑liquid development should be mandatory for responsible e‑liquid development, regardless of the flavour (tobacco or non‑tobacco). 

Do flavoured EVP have greater abuse liability?

The abuse liability of EVP, also termed their dependence potential, is a measure of the likelihood of a given tobacco or nicotine product to induce dependence among users. The main factors used to determine the abuse liability of tobacco and nicotine products are nicotine pharmacokinetics, principally the rate and extent of nicotine absorption into the blood [123], and subjective effects such as craving/withdrawal relief, satisfaction, liking, and intent to use again [124,125]. While EVP should ideally have an abuse liability no greater than that of conventional cigarettes, it is postulated that possessing at least some degree of abuse liability is necessary for the ability of a novel nicotine product to displace cigarettes and lead to complete switching [2,17,18,124,126]. Should EVP flavours increase the abuse liability of EVP, this may be supportive of the overall population‑level THR [126]. However, EVP with too high an abuse liability may have a negative impact, by generating an initiation and/or dependence risk among non‑users of nicotine, and particularly among susceptible populations such as youth [17,18].

A number of recent studies have assessed nicotine pharmacokinetics and/or subjective effects in clinical laboratory studies to determine whether flavours affect abuse liability. In studies with various pod‑system EVP, including JUUL, Vuse Solo, and BIDI sticks, only minor differences were observed between flavours for both nicotine pharmacokinetics and subjective effects [127-130], although the evidence for a lack of a flavour effect is not unequivocal [131]. However, when taking the literature as a whole into account, and when considering that while flavours may influence initial appeal and other subjective effects, the speed and magnitude of nicotine delivery is likely to be the predominant factor in causing dependence [123], it is most likely that EVP flavours do not impact abuse liability to any significant degree. This conclusion is supported by studies on open‑system EVP [132] as well as cross‑sectional population‑level assessments of the impact of flavours on EVP dependence [133,134]. However, some human studies using different methodologies, such as online purchase task measures or assessing perceptions, have reported flavour effects [135,136], while in animal studies, the impact of flavours on nicotine self‑administration (a marker for abuse liability), various findings of no effect or small effects have been reported [137]. Overall, though, regardless of whether flavour effects have been reported or not for EVP, there is perhaps greater consensus that even in the presence of flavours, the abuse liability of EVP is lower, and often much lower, than that of conventional cigarettes [124,128,132,138,139]. Future research in this area needs to consider interactions between EVP flavours and other characteristics, such as nicotine concentration [126,140,141], as well as baseline characteristics at the point of EVP initiation, such as menthol or non‑menthol cigarette smoking status, which can influence the relative appeal of tobacco‑flavoured or non‑tobacco‑flavoured EVP [126]. Additionally, the downstream impact of EVP abuse liability with regard to switching potential among adult smokers also needs further assessment [142] in order to facilitate a greater understanding of the link between EVP flavours, abuse potential, and THR.

Do flavours play a role in switching behaviour among adult smokers?

It has been posited that restricting access to non‑tobacco-flavoured EVP could impede smoking cessation among adults [143] and, therefore, have a detrimental impact on public health. Central to supporting this argument would be evidence that non‑tobacco-flavoured EVP are preferred by adult smokers and also evidence that they facilitate smoking cessation to a greater degree than EVP which are tobacco‑flavoured. The evidence for a supportive role of flavoured EVP in supporting smoking cessation over and above that of tobacco-flavoured EVP is not unequivocal [144-150]. However, a number of studies have provided supporting evidence that non‑tobacco-flavoured EVP,s including mint/menthol, fruit, dessert/pastry/bakery, and candy/chocolate sweet flavours, convey a greater switching benefit in terms of either cigarette smoking reductions or complete switching [151-159] and may reduce cigarette dependence [159]. These divergent findings regarding the ability of flavoured EVP to support switching may reflect the high degree of heterogeneity between studies, and it has been suggested that further randomised controlled studies are required to delineate the presence or absence of a switching benefit of flavoured EVP [150].

It is also of note that fruit‑flavoured EVP were preferred among adults trying to quit smoking [147], and greater preference for both menthol and sweet flavours was associated with lower likelihood of EVP use discontinuation, i.e., these flavours may support continued, long‑term exclusive use and, therefore, prevent relapse back to cigarette smoking [149,160]. Adult EVP users, including both current and former smokers, find non‑tobacco flavours more appealing and satisfying [149,161]. Flavours may play a role in EVP use initiation among current smokers [162,163], and mint and menthol flavours may be favoured by former smokers [163]. Furthermore, some evidence supports that flavour use is increasing among adults [164], and also that migration away from tobacco flavours and towards non‑tobacco flavours among former‑smoking EVP users occurs over time [162,165].

Other considerations regarding flavours include that menthol‑flavoured EVP are favoured by menthol smokers and are more effective at reducing smoking urges in this cohort [166,167], suggesting that menthol availability may be of importance for specific segments of the smoking population. This may be particularly pertinent in reducing the disproportionate tobacco‑related health disparities among certain race/ethnic groups, including Black and Hispanic/Latinx adult smokers, among whom use of menthol cigarettes is more prevalent [167]. Mint/menthol flavours may not only support menthol smokers in switching though with some evidence suggesting support for non‑menthol cigarette smokers [168]. However, the recent findings that menthol or other flavours had no impact on subjective effects, behavioural intentions and craving/withdrawal compared with tobacco flavour [130,169] suggest that further research is necessary in this area to fully delineate the role of flavours in supporting switching among both menthol and non‑menthol cigarette smokers.

Overall, it has been suggested that maintaining flavour diversity is an important consideration when putting in place regulatory frameworks that aim to maximise the switching benefit for adult smokers [152,158,163,169]. However, in order to determine the true THR potential of flavoured EVP, the impact of non‑tobacco-flavoured compared with tobacco-flavoured EVP on smoking initiation or reinitiation among current non‑smokers may require further investigation [144,153].

Do flavours increase unintended use?

As described above, the availability of non‑tobacco-flavoured EVP may contribute to THR by supporting smokers in switching away from cigarette smoking, particularly in those smokers for whom flavours are appealing and satisfying. Use by those for whom they are not intended, such as youth and never‑smokers, may, however, be detrimental to overall population‑level health [17,18]. While adult smokers switching to exclusive EVP use undoubtedly reduces risk compared with continued smoking [11,20,22], any use of EVP among those who are either never smokers or naïve to nicotine is rightly considered to be more harmful than no nicotine use at all. Balancing these two competing impacts on public health can be challenging, but as demonstrated in the US with the recent awarding of marketing-granted orders for menthol‑flavoured NJOY and JUUL EVP [47,170], this challenge is not insurmountable. There is an additional concern that EVP may act as a gateway into nicotine use and subsequently to cigarette smoking among never‑smokers, including youth [171-175]. However, many studies have shown that no such gateway exists, and evidence which appears to support the gateway hypothesis may instead be explained by confounding and the presence of a 'common liability', a genetic predisposition to risk-taking, or a higher behavioural impulsivity among certain individuals [176-185]. That said, maximisation of the THR potential of EVP requires assessing unintended use with a view to minimising it. Various aspects of EVP flavours in relation to unintended use have been examined and reported upon, including the higher likelihood of multiple flavour use and switching between flavours among youth [186], the lower likelihood of EVP cessation and therefore an increased likelihood of long‑term use [187], and an increased willingness to try EVP among youth [188]. However, despite the widespread availability of flavoured EVP, the use of EVP by youth is currently at low levels and declining [189]. Furthermore, although the majority of youth who use EVP use flavoured products [189], flavour availability is not a strong reason for use. Other factors such as experimentation, family or friends using them, curiosity, feeling anxious, stressed or depressed, to get a buzz from nicotine, or to reduce harms to oneself and others around them, are commonly expressed as either equal or stronger drivers than flavours [190-193]. Some studies do suggest that banning flavours may reduce the appeal of EVP and reduce youth use [194-196]. However, other studies did not produce findings that concur with this view. One study using a life‑sized model convenience store found that flavour bans did not affect intentions to use menthol/mint or sweet flavoured EVP. Conversely, removal of mint/menthol and sweet flavoured products increased intentions to use tobacco‑flavoured products among those who had already started using EVP [197]. Hypothetical flavour bans may lead to continued EVP use (presumably of non‑restricted flavours) and may further lead to the highly undesirable outcome of continued smoking or switching to smoking, exacerbating population health harms [198]. We can also draw upon real‑world examples of flavour bans, for example, following the US Food and Drug Administration (FDA) 2020 enforcement action against flavoured pod‑system EVP, which effectively banned the sale of cartridge‑based (pod‑system) EVP with flavours other than menthol or tobacco [199]. This led to both flavour and device‑type switching, including among youth [200-202]. Consumption patterns, assessed using nicotine exposure biomarker data from the US Population Assessment of Tobacco and Health (PATH) study, were not impacted by the EVP flavour used among youth [203], a finding that counteracts the argument that flavours enhance attractiveness and increase nicotine intake/consumption [44]. Other data suggest that youth use of flavours is experimental [204,205] and such experimentation may not translate into long‑term EVP use.

Overall, one of the greatest challenges of maximising the THR potential of EVP is that both adults and youth may have a preference for flavoured EVP and find them appealing [206-208]. This means that any regulatory measure to minimise unintended use by restricting the availability of flavoured EVP will likely have the unintended and detrimental consequence of removing products from markets which otherwise would have helped smokers switch [209], and consequently curtailing the declines in cigarette smoking prevalence seen in recent years [35,36].

Are flavour bans effective?

Should EVP flavours be restricted, it is important to consider the consequences, both intended and unintended and on smokers and non‑users of tobacco products, in order to determine the population‑level impact on public health of such action. Many proposals by various bodies have been put forward to restrict or ban the use of non‑tobacco flavours in EVP [43-45,51], which variously suggest that such action will protect young people from harms associated with nicotine use by reducing EVP appeal and attractiveness [43,210,211], as well as mitigating toxicological concerns [43,44]. What these proposals often do not take into account, however, is the potential impact on adult smokers and former smokers, who, despite suggestions otherwise [43], are the predominant users of EVP [74,75]. These proposals also do not often take into account whether there is evidence base for flavour restrictions having the desired effect.

Banning non‑tobacco EVP flavours has been suggested to increase cigarette sales and overall tobacco use [196,212], with one study in the US state of Minnesota finding that youth use of any tobacco product rose by up to 45% following implementation of tobacco product flavour restrictions [196]. In the US state of New York, a non‑tobacco flavour ban failed to prevent more than 95% of youth EVP users from continuing to use non‑tobacco-flavoured EVP [213]. This is suggestive of the development of an illicit trade in non‑tobacco-flavoured EVP following this flavour ban, which may arise from retailer non‑compliance and/or users procuring banned EVP from other physical and online sources, both legal and illegal. Many studies have either found evidence for, or suggested the development or presence of, an illicit trade for non‑tobacco-flavoured EVP following flavour bans [209,214-224]. The availability of illicit products not only undermines the intended purpose of any regulation but also has the potential to increase health risks to users. In this regard, aerosol from ‘aftermarket’ JUUL pods (i.e., those not manufactured by JUUL but which are compatible with the JUUL device) contains higher levels of carbonyl compounds and reactive oxygen species than those pods made by the manufacturer [225]. If flavour restrictions were specific for specific device types (e.g. pod‑system EVP) or flavours, some evidence suggests the users would simply switch to using non‑tobacco flavours in unrestricted devices, i.e., products which fall outside of regulatory oversight [223,226-231] or ‘migrate’ to using non‑restricted flavours [200,227,229]. Additionally, there is some evidence that following flavour bans, small proportions of users may mix flavoured e‑liquids themselves [216,222,223], which would likely be of toxicological concern and increase potential risks to health of EVP use [232].

While some evidence suggests that EVP use may fall as a consequence of flavour restrictions [223,226,228] or that such bans would encourage at least some users to quit EVP use [209,215], other evidence suggests that flavour restrictions are perhaps largely ineffective. In the US, following the FDA’s 2020 change in enforcement discretion [199], EVP use overall was largely unaffected, among both youth and young adults [230,231,233]. Other studies have provided evidence that following implementation of restrictions, cigarette sales may increase and many EVP users may switch to, or continue, smoking [198,209,210,215,217,221,222,227,230], although some evidence suggests that switching to smoking would not necessarily occur [229,234]. There is also a concern that banning flavours could make smoking more appealing than EVP use [222], especially among youth who are more prone to partake in risky behaviours [235] which could facilitate smoking initiation or reinitiation [236]. This risk of increasing smoking rates and, therefore, overall population level health has been proposed to outweigh the potential benefits to public health of banning EVP flavours [217].

What is the overall population-level impact of EVP flavour restrictions?

**Figure 2:**
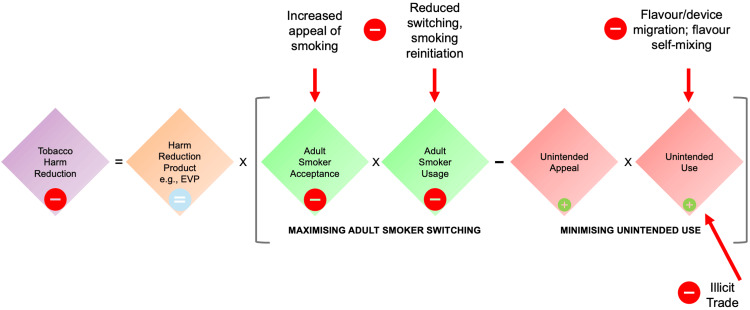
Potential impact of flavour restrictions on electronic vaping products EVP: electronic vaping products While flavour bans may be effective in reducing appeal and use by those for which EVP are not intended, this effect is likely to be only partial and would be offset by behavioural changes among unintended users (e.g. flavour and/or device migration, self‑mixing of flavours, and obtaining EVP through illicit sources). It has also been proposed that the intended impact would be far outweighed, at the population level, by unintended and deleterious effects on adult smokers [198] such as reduced appeal of EVP and therefore reduced switching rates, leading to smoking initiation or reinitiation and increased cigarette consumption

The main findings of this review, placed into the context of whether implementing regulatory restrictions on non‑tobacco-flavoured EVP would be supportive of, or detrimental to, THR and overall population‑level public health, are presented in Figure 2. Generally speaking, there is no firm evidence that non‑tobacco flavours are of risk to health, either in absolute terms or relative to tobacco flavours. While some studies have also shown that flavoured EVP may release potential toxicants into the aerosol, including metals and aldehydes, such findings are not meaningful unless the data arising from such studies are used as inputs into toxicological QRAs. This view should not be taken as meaning that all flavours are safe, and certainly some ingredients which have historically been found in flavoured EVP, such as diacetyl, are of concern and should not be present in EVP liquids which adhere to regulatory standards or which have been developed following rigorous toxicological risk assessment protocols. Such standardised toxicological QRA procedures are essential to minimise potential harms associated with EVP use, and manufacturers should take responsibility and ensure that such procedures are incorporated into product development. These procedures should take into account the route and degree of exposure, during normal and perhaps intense use, and be informed by studies which have collected aerosol chemistry data or exposed biological materials to EVP aerosol which has been generated in a manner which reflects actual use. This includes not only replicating human puffing topography but also using device settings and including flavouring ingredients at levels which accurately replicate those used by EVP users. Additionally, the use of appropriate control or reference exposures is fundamental to understanding both absolute and relative risk, taking into account that the vast majority of EVP users are former smokers.

There is also little evidence that non‑tobacco-flavoured EVP possess a greater abuse liability than those which are tobacco-flavoured. What is more certain, however, is that restricting flavour choice will have a negative impact on the target population, adult smokers looking to switch to a potentially less harmful alternative to cigarettes [163]. Restricting flavours will reduce both EVP acceptance and use among adult smokers. Strong evidence supports that such regulatory action will potentially even increase the appeal of smoking, reduce switching, and encourage either smoking initiation or reinitiation, thereby creating a gateway into smoking by only allowing tobacco flavours and encouraging experimenting with the real tobacco taste from smoking cigarettes and burning tobacco. Good evidence also suggests that flavour bans have had effects opposite to those intended, including increased cigarette sales and overall tobacco product use in adults as well as youth. Such effects are undoubtedly detrimental to THR, leading to either a slowing in the declines in cigarette smoking seen in many countries, which is associated with EVP use [35], and potentially leading to a reversal in this population health‑promoting trend and an increase in smoking attributable mortality [143].

Evidence supporting an overall population‑health benefit of EVP flavour restrictions is weak. Little evidence supports that reducing unintended use, including among youth, is a consequence of flavour restrictions. Conversely, many studies have provided evidence for the unintended and detrimental effects of such action. These include consumers adopting behaviour changes which mitigate the effectiveness of such regulation, such as migration towards non‑restricted flavours and/or EVP device types, self‑mixing of EVP liquids to include non‑tobacco flavours which are of risk to health, or accessing EVP through physical and online sources, both licit and illicit. Such behaviour change not only undermines the regulatory intention, but it does so at the expense of the intended behaviour change, switching among adult smokers. Other responses to regulation may also occur, such as the inclusion by manufacturers of non‑restricted flavour ingredients into EVP liquids.

Given the competing balances of THR, and trying to maximise adult switching while minimising unintended use, the question becomes how do we best achieve this? How do we continue to tap into the strong THR potential of EVP and support the continued decline of cigarette smoking, while protecting against youth use? This is a hugely important question, given that an estimated seven million smokers die each year from smoking‑attributable disease [1]. The answer is not to simply restrict flavour diversity and choice for adult smokers in order to reduce youth use. The existing evidence suggests that this would be ineffectual in meeting its intended goal and runs the risk of derailing recent advances in reducing smoking prevalence and, subsequently, morbidity and mortality. The setting of regulatory policy should bear in mind that smoking‑related disease and death are disproportionate and concentrated among certain race/ethnic groups, as well as the older adult population, and that current tobacco control measures have not proven successful in reducing the absolute number of smokers globally. Regulatory policy should also take into account the vast potential of EVP to help adult smokers switch to less harmful alternatives to smoking [237]. This includes maintaining flavour diversity, a supporting factor in helping adult smokers switch. The recent rises in youth EVP use, which are perhaps experimental and not sustained, can be tackled more effectively than by banning flavours, which is an overly simple solution to a much more complicated problem of public health. Greater enforcement of existing youth protection regulations is one part of a potential solution [238], which could include licensing schemes to restrict where EVP can be sold. Additional gains could be made by prohibiting products, and their marketing materials, packaging and flavour descriptors, which are overtly attractive to youth. This could be achieved, perhaps, through the implementation of responsible marketing standards. While these are more complex solutions than flavour prohibition, they would help to protect smokers’ access to the diverse range of products that they currently have and which likely underpins the association of increased EVP use prevalence with smoking declines, while preventing youth access. Such approaches to EVP regulation would protect those who would benefit most from using EVP, but not to the detriment of those who would not. Hence, the overall end goal of eliminating smoking‑related death and disease could remain in sight. 

## Conclusions

In summary, the use of non-tobacco flavours in EVP is a complex issue in which many competing factors play a role in determining the overall impact on population-level health. When setting policy, regulators should take all these factors into account, including the use of flavoured EVP by adult smokers as a means by which to quit smoking, and maintaining flavour diversity may be supportive of reducing smoking prevalence and improving public health. 
